# Wildflower plantings promote blue orchard bee, *Osmia lignaria* (Hymenoptera: Megachilidae), reproduction in California almond orchards

**DOI:** 10.1002/ece3.5952

**Published:** 2020-02-25

**Authors:** Natalie K. Boyle, Derek R. Artz, Ola Lundin, Kimiora Ward, Devon Picklum, Gordon I. Wardell, Neal M. Williams, Theresa L. Pitts‐Singer

**Affiliations:** ^1^ USDA ARS Pollinating Insects Research Unit Logan UT USA; ^2^ Department of Entomology Center for Pollinator Research The Pennsylvania State University University Park PA USA; ^3^ Department of Entomology and Nematology University of California Davis CA USA; ^4^ Department of Ecology Swedish University of Agricultural Sciences Uppsala Sweden; ^5^ Department of Biology University of Nevada Reno NV USA; ^6^ Private Consultant Paso Robles CA USA

**Keywords:** floral resources, managed pollinators, pollen, *Prunus dulcis*

## Abstract

Concerns over the availability of honeybees (*Apis mellifera* L.) to meet pollination demands have elicited interest in alternative pollinators to mitigate pressures on the commercial beekeeping industry. The blue orchard bee, *Osmia lignaria* (Say), is a commercially available native bee that can be employed as a copollinator with, or alternative pollinator to, honeybees in orchards. To date, their successful implementation in agriculture has been limited by poor recovery of bee progeny for use during the next spring. This lack of reproductive success may be tied to an inadequate diversity and abundance of alternative floral resources during the foraging period. Managed, supplementary wildflower plantings may promote *O. lignaria* reproduction in California almond orchards. Three wildflower plantings were installed and maintained along orchard edges to supplement bee forage. Plantings were seeded with native wildflower species that overlapped with and extended beyond almond bloom. We measured bee visitation to planted wildflowers, bee reproduction, and progeny outcomes across orchard blocks at variable distances from wildflower plantings during 2015 and 2016. Pollen provision composition was also determined to confirm *O. lignaria* wildflower pollen use. *Osmia lignaria* were frequently observed visiting wildflower plantings during, and after, almond bloom. Most *O. lignaria* nesting occurred at orchard edges. The greatest recovery of progeny occurred along the orchard edges having the closest proximity (80 m) to managed wildflower plantings versus edges farther away. After almond bloom, *O. lignaria* nesting closest to the wildflower plantings collected 72% of their pollen from *Phacelia* spp., which supplied 96% of the managed floral area. *Phacelia* spp. pollen collection declined with distance from the plantings, but still reached 17% 800 m into the orchard. This study highlights the importance of landscape context and proximity to supplementary floral resources in promoting the propagation of solitary bees as alternative managed pollinators in commercial agriculture.

## Graphical abstract

The blue orchard bee, *Osmia lignaria* (Say), is a commercially available native bee that can be employed as a copollinator with, or alternative pollinator to, honeybees in commercial orchards. However, their success is limited by poor progeny recovery in managed systems. Through the installation and maintenance of coblooming alternative floral resources near orchards, we were able to support higher reproduction of blue orchard bees in CA almonds; their success was correlated with landscape context (orchard edges vs. interiors) and relative proximity to the alternative forage.

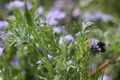

## 1 INTRODUCTION

The California almond (*Prunus dulcis* Mill.) industry relies heavily on the availability of honeybees (*Apis mellifera* L.; Hymenoptera: Apidae) to meet the pollination demands of their orchards (Traynor, 2017). These demands have grown in recent years as the amount of almond‐bearing acreage now exceeds 470,000 ha (CDFA, 2019), requiring over two million honeybee hives annually during bloom (Goodrich & Goodhue, 2016), and accounting for 73% of the U.S. honeybee population (as of January 2017; Goodrich, 2018). Consequently, it has become increasingly more difficult for commercial beekeepers to meet the pollination demands of the industry (Aizen & Harder, 2009; Seitz et al., 2016; Ward, Whyte, & James, 2010), which is compounded by persistent stressors impacting honeybee health and survival (vanEngelsdorp et al., 2009). Incorporating Integrated Crop Pollination strategies that supplement orchard pollination with alternative bee species may become necessary to bridge the widening gap between honeybee colony supply and demand (Bosch & Kemp, 2001; Isaacs et al., 2017; Wesselingh, 2007).

The blue orchard bee, *Osmia lignaria* Say (Hymenoptera: Megachilidae), native to North America (Rust, 1974), has been implemented effectively as a pollinator of commercially managed orchard crops, including apples, cherries, and almonds (Bosch & Kemp, 1999; Sheffield, 2014; Torchio, 1979, 1985). When employed in commercial almond orchards, research shows that *O. lignaria* copollination with honeybees in almond orchards significantly increases fruit set versus when either pollinator is implemented alone (Brittain, Williams, Kremen, & Klein, 2013; Pitts‐Singer, Artz, Peterson, Boyle, & Wardell, 2018). These results hold true in both semifield and open‐field studies ranging from 20 × 13 × 3 m enclosed cages (Brittain et al., 2013) and up to 4‐ha tracts of open almond orchard (Pitts‐Singer et al., 2018). Presently, the greatest challenge for the successful implementation of this alternative pollinator is their limited supply, coupled with their high cost. Sustainable in‐orchard reproduction of *O. lignaria* in commercial orchards for use during the following year is not always achieved (Artz, Allan, Wardell, & Pitts‐Singer, 2013, 2014). Except for the occasion when in‐orchard progeny recovery exceeds the number of *O. lignaria* initially released (Boyle & Pitts‐Singer, 2017; Pitts‐Singer et al., 2018), most *O. lignaria* currently available for distribution are captured from natural environments, which may have repercussions on native populations and their contributed ecosystem services to wildlands (Tepedino & Nielson, 2017). Additionally, trapping bees is labor‐intensive, and management practices for the processing and cleaning of cocoons to eliminate pests and diseases can be costly. Therefore, initial retail costs of *O.* *lignaria* for commercial pollination exceed costs associated with hiring contracted honeybee pollination services at recommended stocking rates (Koh, Lonsdorf, Artz, Pitts‐Singer, & Ricketts, 2018). Undoubtedly, in‐orchard management practices must improve to support higher rates of *O. lignaria* reproduction for this species to become a viable alternative or supplement to honeybee almond pollination.

Almond blossoms are only available to foraging bees for 2–3 weeks of the year, which does not fully accommodate the 4–6‐week life span of foraging *O.* *lignaria*. This limits the foraging period of *O. lignaria* in commercial orchards, where intense chemical control of weeds and other vegetation prevents pollinator access to supplementary floral resources that may extend their reproductive season and improve their overall nutrition. Nutritional limitation is one proposed explanation for the widespread decline of pollinator populations, particularly where monocultures dominate the landscape and offer only one or two mass floral resources for foraging bees (Brodschneider & Carlsheim, 2010). Further, nutritional limitation can immunocompromise bees, making them more susceptible to parasites and pathogens (DeGrandi‐Hoffman & Chen, 2015) and slow or inhibit immature solitary bee development (Praz, Müller, & Dorn, 2008).

Our objective was to determine the impact of managed wildflower plantings installed adjacent to commercial almond orchards on *O. lignaria* reproductive success. In total, 28.8 ha of commercial almond orchards were supplemented with managed *O. lignaria*, at variable distances from managed floral plantings. We hypothesized that access and proximity to alternative floral resources would improve *O. lignaria* nesting and reproduction during (and following) almond bloom. Three plots of previously fallow land, alongside a commercial almond orchard, were seeded with wildflower species known to overlap with (and extend beyond) almond bloom, providing diverse and extended floral resources for nesting *O. lignaria* through 2015 and 2016. Visitation to wildflower plantings, rates of bee reproduction, progeny outcomes, and pollen composition of representative provision masses were compared across six discrete distances (or “zones”) from the maintained wildflower plantings to verify the use of alternative floral resources.

## 2 MATERIALS AND METHODS

### 2.1 Experimental layout and *O. lignaria* management

**Figure 1:**
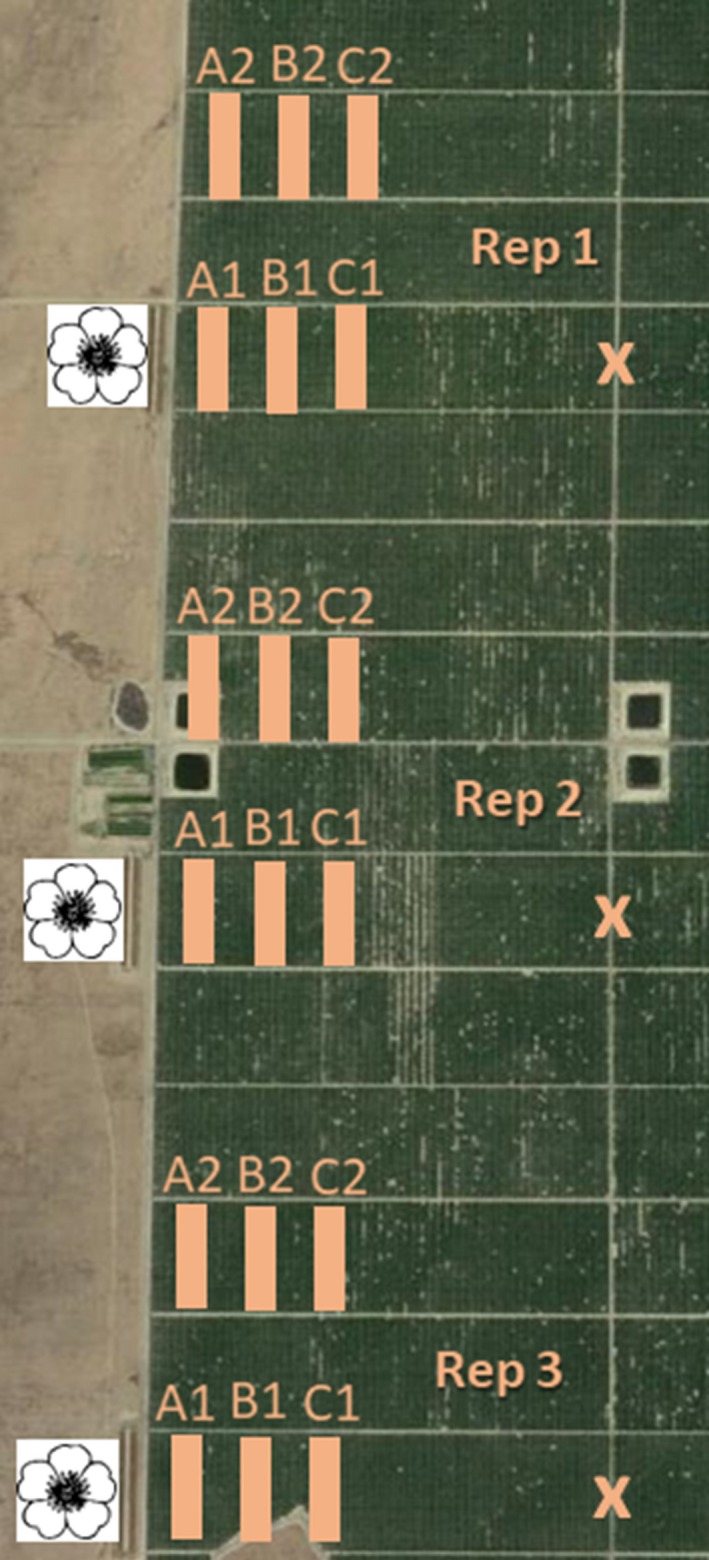
Aerial view of experimental orchards in Lost Hills, Kern Co., California. Three wildflower strips (indicated by white floral squares) were planted and maintained west of orchards through 2015 and 2016 almond bloom. *Osmia lignaria* were released into one of six zones (orange rectangles; lettered A1–C2), 1.6 ha in size, with associated nesting materials. “X” denotes areas of limited bee releases to evaluate pollen provision composition at extreme distances (800 m) from the wildflower plot (2016 only)

This study was conducted across a 2.12 km^2^ swath of commercial almond orchards near Lost Hills, Kern County, CA (Figure 1), in the southern Central Valley during 2015 and 2016. The surrounding landscape was dominated by neighboring almond orchards and fallow, unmanaged land. Experimental orchards were owned and managed by a single operator and consisted of Nonpareil almond trees flanked by alternating pollinizer rows of Monterey and Wood Colony varieties. Rows were oriented north‐to‐south and divided into contiguous 16.2‐ha blocks separated by maintained 4‐m‐wide dirt access roads, or “beeways.” Each 16.2‐ha block included 109 rows of almond trees, with 30–32 trees per row. Honeybee hives were placed in groups of 24 at three equally spaced locations along each beeway, resulting in an in‐orchard stocking rate of approximately five hives per hectare (the typical recommendation for almond pollination; Goodrich & Goodhue, 2016).

Three pairs of 16.2‐ha orchard blocks were selected within the experimental orchards to receive managed *O. lignaria* populations in addition to the standard five honeybee hives per hectare. Along the southwestern edge of each paired orchard block, a 0.48‐ha wildflower planting was established with native wildflower species that overlap with and extend beyond almond bloom (Figure 1; Table 1). The northwestern edge of the other orchard block in each pair was left as fallow land. Wildflower seeds were planted in autumn 2014 (early November) and 2015 (early October). Plantings were irrigated after seeding until March (2015) or February (2016) to promote establishment. Further, flowering details of the plantings, including reports of floral area over time, are reported in Lundin et al. (2017). In summary, the plantings started flowering slightly before almonds, peaked during almond bloom, and extended ca. 4 weeks beyond almond bloom. *Phacelia ciliata* dominated the wildflower plantings in both years and provided 96% of the floral area (Lundin et al., 2017). Orchard understories were kept bare via chemical control of weeds and vegetation, so that almost no competing floral resources other than those provided by almond trees and the wildflower plantings were available to foraging bees. Some wild mustard, grasses, and other drought‐tolerant pollen sources appeared during late almond bloom in fallow land surrounding the wildflower plantings, although no species overlap between weeds and managed plantings occurred (Lundin et al., 2017).

**Table 1:** Wildflower planting species composition and associated bloom windows

Species	Common name	Bloom season
*Calandrinia ciliata*	Redmaids	February–May
*Collinsia heterophylla*	Chinese houses	February–April
*Eschscholzia californica*	California poppy	April–July
*Nemophila maculata*	Five spot	February–April
*Nemophila menziesii*	Baby blue eyes	March–June
*Phacelia capanularia*	Desertbells	February–April
*Phacelia ciliata*	Great valley Phacelia	February–May

**Figure 2:**
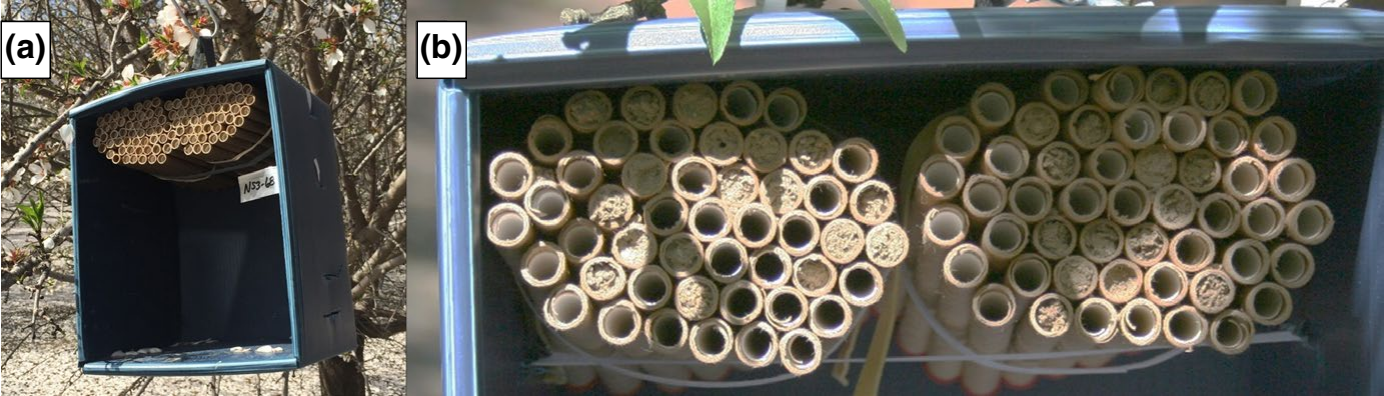
Materials for managing large populations of *Osmia lignaria* for commercial pollination include (a) corrugated plastic nest boxes, complete with (b) cardboard nesting cavities, lined with straw paper inserts for *O. lignaria* nesting. Nest completion was tracked over time by counting mud plugs (pictured) at each nest box

*Osmia lignaria* were released in six equally spaced 1.6‐ha orchard regions within paired 16.2‐ha blocks. These orchard regions, or “zones,” varied in their proximity to the wildflower plantings and orchard edges across the three replicates, ranging from 80 m (zone A1) to 580 m (zone C3) (Figure 1; Table 2). Within each zone, 40 nest boxes were distributed uniformly, in accordance with best management practices (Bosch & Kemp, 2001; Koh et al., 2018). One‐hundred nesting tunnels were installed in each nest box to meet the recommended density of at least two nesting tunnels per female released (Bosch & Kemp, 2001). Nest boxes were folded corrugated plastic boxes (21.5 × 20 × 25.5 cm; Figure 2a). Nesting tunnels were cardboard tubes (7.2 mm diameter × 15.2 cm deep), each lined with a glassine paper straw insert (Figure 2b). Each nest box received an application of bee attractant (Pitts‐Singer et al., 2016) prior to their deployment to improve retention.

**Table 2:** Average distance of nesting boxes to wildflower plantings, by zone (2015 and 2016)

Zone	Mean distance (m) from wildflower planting
A1	80
B1	240
C1	400
A2	410
B2	475
C2	580

In January 2015 and 2016, cocooned *O. lignaria* adults were obtained collectively from various suppliers from Idaho, Oregon, Utah, and Washington. Bees were released from centrally located emergence boxes in each zone across all three experimental orchard replicates (18 total orchard release sites) using standard practices outlined in Appendix S1. We released 750 female and 1,200 male *O. lignaria* per ha (or 1,200 females and 1,920 males per zone).

Both 2015 and 2016 were drought years in California, which limited natural and reliable access to wetted soil for *O. lignaria* nest‐building. In 2016, water trucks delivered water to soil along beeways between experimental orchard blocks, twice per week, to facilitate female *O. lignaria* construction of mud partitions during nest‐building. The supplemental water maintained moist mud along orchard edges of the beeways throughout the nesting period; such accommodations were not made in 2015.

Nest boxes were left in the orchard until late April in 2015 to allow larval development to late instars prior to their removal (Bosch & Kemp, 2001). In 2016, nest boxes were removed earlier (mid‐April), due to the scheduled destruction of experimental orchards prior to harvest. Upon their removal from the orchard, nest boxes were held in a warehouse at ambient temperature until mid‐August for both years. During this time, offspring continued their development to adulthood in enclosed cocoons, the developmental stage at which they enter winter diapause. Upon reaching the adult stage, bees were introduced to a 4°C incubator for overwintering and held until the following spring.

### 2.2 *Osmia lignaria* visitation

*Osmia lignaria* visitation to wildflower plantings and in fallow plots was monitored at five time points in 2015 and 2016 to confirm their use of planted floral resources. Time points selected were representative of peak almond bloom, late bloom, 1 week following bloom, 2–3 weeks following bloom and 4 weeks following bloom. These coincided with assessments of floral area at wildflower plots and within adjacent, fallow fields (as reported in Lundin et al., 2017). *Osmia lignaria* visitation to all plots wereas recorded along two 50‐m transects at each time point, using methods described in Lundin et al. (2017) and detailed in Appendix S2. Visitation rates, summed across season, were compared using a generalized linear mixed model with a negative binomial distribution and log link function via PROC GLIMMIX in SAS 9.2 (SAS Institute, 2008) with treatment (wildflower or control) as a fixed factor and the replicate pair of wildflower and control plots as a random factor. Each year was analyzed separately.

### 2.3 Nesting over time

*Osmia lignaria* nesting was monitored regularly throughout 2015 and 2016 foraging periods to record nest completion over time. Starting 1 week after their release in the orchards, we recorded nest completion by taking photographs of all nest boxes every 5–7 days until late March (both years). Completed nests were discernable in photographs by the presence of a mud plug at the terminal end of nesting tunnels (Figure 2b). The number of plugged tunnels was recorded for every nest box six times in 2015 and eight times in 2016. In 2016, we released additional bees at extreme “long‐distance” nest sites, installed along the eastern edge of experimental orchard blocks, 800 m from managed wildflower plantings (Figure 1). Long‐distance nest boxes were also photographed (further details are reported in Appendix S3).

Differences in nesting by zone and over time were assessed via PROC GLIMMIX in SAS version 9.2. The model utilized a normal distribution with an identity link function for additive completed nests over time. Zone and time were fixed factors, and replicate was specified as a random effect in the model. Interzone comparisons were conducted via Tukey pairwise comparisons. Independent analyses were conducted for each year due to variation in bee management practices.

### 2.4 Reproduction and progeny outcomes

In August 2015 and 2016, total bee reproduction from the nesting tunnels was assessed using digital X‐radiography (6‐s exposure at 22 kVp, Faxitron 43804N; Faxitron Bioptics). The resulting images provided a full census of bee reproduction and progeny outcomes. Metrics from the census included the number of cells with viable progeny, proportional mortality of cells, female‐to‐male sex ratio, average cells produced per tunnel, proportion of bees that died during development, proportion of cells occupied by parasites and/or scavengers, and the proportion pollen ball, which are cells with uneaten provisions on which no progeny had developed (Boyle & Pitts‐Singer, 2017; Pitts‐Singer et al., 2018). Sex, cause, and stage of death were determined by evaluating the relative size, position, and contents of cocoons within a given nest (pictured in Boyle & Pitts‐Singer, 2017). Because weather and specific strategies for in‐orchard management varied by year, cross‐year comparisons were not conducted. One‐way ANOVAs with replicate as a random factor were performed to evaluate differences in *O. lignaria* reproduction by zone in SAS PROC ANOVA. Tukey's multiple comparisons were conducted to distinguish differences between zones.

### 2.5 Pollen provision composition

To examine the extent *O. lignaria* used floral resources from the wildflower plantings, samples of provision masses from individual nest cells were collected, and individual pollen grains were identified to genus using a light microscope. Species determinations were made from a reference collection of pollen from each species in the wildflower plantings, as well as those occurring in surrounding, fallow landscapes.

Fifteen completed nests were collected in rows at distances 85, 260, and 435 m from the wildflower plots twice in 2016 within zones A1–C1 only (Figure 1). Nests were first sampled at the onset of petal fall on 8 March 2016, at which time all completed, “plugged” nests were painted with blue acrylic paint over the terminal mud plug throughout the selected rows. On 26 March 2016, about 1 week after almond bloom, we returned to the same orchard rows and collected 15 newly plugged, unpainted nests.

Pollen composition was determined only from the terminal (most recently completed) provision of each individual nest sampled. Samples were collected by using a razor blade to make an “X” incision on the paper straw insert to access the provision. Then, using a different toothpick for each nest, a small sample of the provision was removed, placed onto a microscope slide, and stained with fuchsin jelly under a coverslip. Razor blades were thoroughly cleaned between the handling of each nest.

A minimum of 125 pollen grains were identified from each provision sample, which is similar to other count parameters described in O'Neill, O'Neill, Blodgett, and Fultz (2004), Santos et al. (2013), and Lau et al. (2019). From each pollen slide, photographs were taken under 40× magnification, and all pollen grains within the field of view were counted and identified. For photographs with fewer than 125 pollen grains, a second photograph was recorded from elsewhere on the slide, and all grains were counted and identified within that photograph as well. Due to the variable number of pollen grains counted within each sample, our findings are presented as proportional data, rather than raw counts. The pollen composition of provisions from completed long‐distance tunnels was also determined using these same methods. Results for long‐distance nests are reported in Appendix S3.

Pollen composition of provisions was evaluated first using proportions of pollen grain counts (number of grains per species), and again after adjusting count data to accommodate for differences in pollen grain volume (volume‐adjusted data methods and results are reported in Appendix S4. Because the dominant wildflower species represented in sampled provisions were almond and *Phacelia* spp., individual pollen grains were broadly categorized into one of three groups: “almond,” “*Phacelia* spp.,” and “other.” “Other” pooled all pollen grains identified from plants which were not almond or *Phacelia* spp. This included plants from both managed wildflower plantings and neighboring fallow land. Proportional count data were statistically analyzed using a generalized linear mixed model with a beta distribution and logit link function via PROC GLIMMIX in SAS version 9.2.

## 3 RESULTS

### 3.1 Visitation

**Figure 3:**
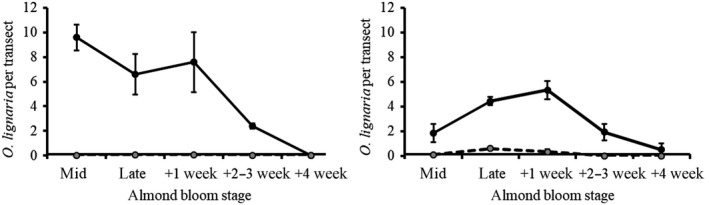
*Osmia lignaria* visitation (counts per 50 m × 1 m transects) in wildflower plantings (solid bars) and neighboring fallow land (dashed lines) during and after almond bloom 2015 (left) and 2016 (right), ±*SEM*

*Osmia lignaria* were observed frequently in the wildflower plantings throughout 2015 and 2016 during and after almond bloom (Figure 3). *Osmia lignaria* visitation was higher in wildflower plantings compared to fallow orchard edges (“control”) in both years (2015: *F*_1,2_ = 49.30, *p* = .020; 2016: *F*_1,2_ = 77.48, *p* = .013).

### 3.2 Nesting

**Figure 4:**
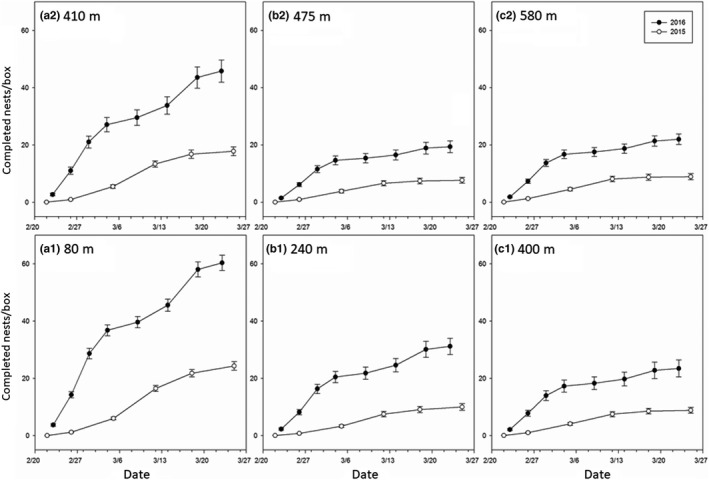
Mean *Osmia lignaria* nest completion over time, ±*SEM*, in 2015 (open circles) and 2016 (closed circles). Zone name (lettered A1–C2; Figure 1) and average distance from wildflower plantings are indicated by the values presented within the panels above. The number of completed nests is per nest box (40 boxes per zone and replicate, each containing 100 tunnels per box). For both years, almond bloomed ended approximately 5 March

**Figure 5:**
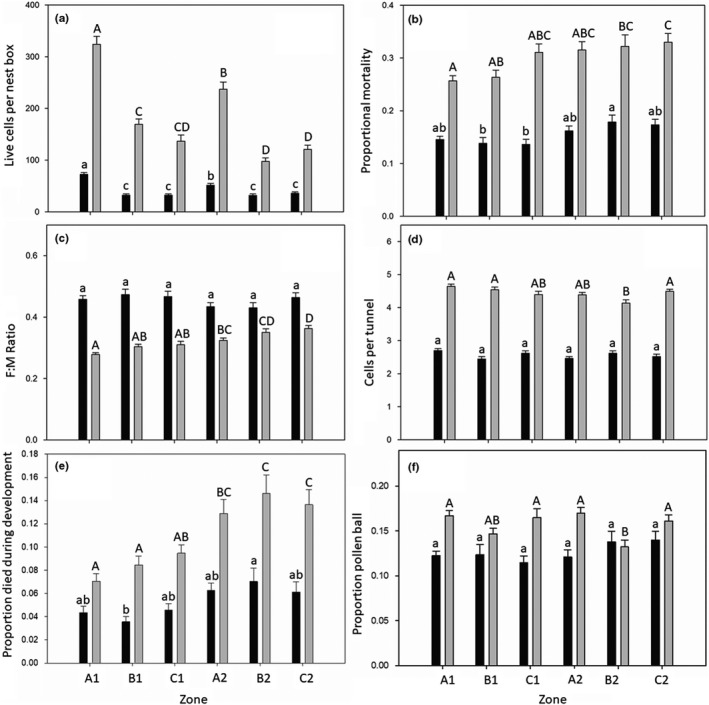
End‐of‐season 2015 (black) and 2016 (gray) *Osmia lignaria* nesting by zone. Results are interpreted from X‐radiography, from a census of all bees recovered, in response to zone (distance from managed floral plantings), ±*SEM*. Presented are (a) average number of live cells recovered per nest box, (b) average proportional mortality (pooled dead immature bees, dead adults, pollen ball, and parasitized cells), (c) female‐to‐male sex ratio, (d) average cells per nesting tunnel, (e) proportion of progeny that died during development (dead immature bees only), and (f) the proportion of cells with pollen ball, in which the provision mass remains uneaten. Due to differences in orchard and bee management in 2015 and 2016, cross‐year comparisons were not made; significance from Tukey multiple comparisons is indicated within years with lowercase (2015) and uppercase (2016) lettering

More nesting occurred, and more cells were produced, in 2016 versus in 2015 (Figures 4 and 5; Tables S1–S5). The relative location of nest blocks in the orchards (by zone), in addition to the time of year, significantly influenced *O. lignaria* nesting in 2015 and 2016 (Table S1). The highest overall nesting occurred at orchard edges (zones A1 and A2), with lower nesting in the orchard interior (Figures 4 and 5; Tables S4 and S5). Despite notable differences in the geographic distances separating “edge” zones A1 (80 m) and A2 (410 m) from the wildflower plantings, no significant difference in nesting was determined for 2015 or 2016 (Table S2). Likewise, no differences were detected for nesting over time for any combination of interior zones (B1–C2) for either year (Table S2).

Across all zones, the greatest rate of *O. lignaria* nest completion was observed consistently between 5 March and 12 March in 2015 and between 26 February and 1 March in 2016 (Figure 4). Nesting continued after almond bloom had ceased in every zone over both years (Figure 4). Nesting at long‐distance sites (Figure S2, Appendix S3) did not continue after bloom, and considerably less nesting occurred overall.

### 3.3 Reproduction and progeny outcomes

Rates of predation and parasitism were low across all zones, accounting for about 3% of the mortality observed in both years (Tables S4 and S5). During both years, more cells were recovered from A1 than in any other zone, while A2 generated the second highest number of live cells recovered (Figure 5a). In 2016, mortality was highest in zones further from the wildflower planting (Figure 5b). In 2015, zone did not impact the female‐to‐male sex ratio of *O. lignaria* progeny (Table S3, Figure 5c). Significantly, more 2016 females were observed in zones with increasing distances from the wildflower planting (Table S5, Figure 5c). Notably, 2016 sex ratio results do not imply greater overall female reproduction away from the wildflower planting, as zones along orchard edges resulted in significantly higher nesting overall (Figures 4 and 5). Significant differences in average cells per tunnel were apparently not tied to distance from wildflower plantings for either year (Figure 5d), and overall, fewer cells per nest were generated in 2015 (2016 mean cells per nest: 4.43 ± 0.08 *SEM*, vs. 2015 mean cells per nest: 2.56 ± 0.07 *SEM*). A greater proportion of 2016 immature bees failed to develop to the adult stage compared to those in 2015 (Figure 5e). While significant, no meaningful trend in developmental mortality could be determined in 2015 by zone (Table S3, Figure 5e). However, in 2016, zones geographically closest to the wildflower plantings experienced significantly less proportional developmental failure than progeny in zones further away (Table S3, Figure 5e). Zone B2 exhibited a significantly lower proportion of cells with pollen ball in 2016 (Table S3, Figure 5f). A similar trend was not observed in 2015 (Table S3).

### 3.4 Pollen provision composition

*Osmia lignaria* frequently used pollen from almond trees and the wildflower plantings across all zones (Table 3 and Table S6). By far, the most abundant pollen grains counted were *Phacelia* spp. and almond grains; any other pollen grains identified were pooled and presented as “other” in Table 3. The plant species most frequently encountered in “other” included managed *Nemophila* spp. (45% of “other” pollen grains; encountered in 45% of all nests), wild grass (30% of “other” pollen grains; encountered in 86% of all nests), and wild *Amsinckia* sp. (24% of “other” pollen grains; encountered in 41% of all nests). *Collinsia heterophylla* was observed infrequently in pollen provisions, and no *E. californica* grains were identified in any sampled provisions.

**Table 3:** Pollen counts presented as a percentage of *Osmia lignaria* pollen provisions located at 85, 260, 435, and 800 m away from managed wildflower plantings at peak and postalmond bloom

	Near		Middle		Far	
	Peak	Post	Peak	Post	Peak	Post
Almond (%)	34.4 ± 7.3	18.0 ± 4.7	46.3 ± 8.1	20.5 ± 5.2	74.4 ± 6.1	25.0 ± 6.0
Phacelia (%)	41.7 ± 7.3	71.8 ± 6.1	31.0 ± 6.4	62.6 ± 7.0	18.5 ± 4.6	58.7 ± 7.3
Other (%)	20.8 ± 4.2	19.7 ± 4.1	16.5 ± 3.6	21.9 ± 4.3	9.0 ± 2.3	19.1 ± 4.0

Note

The most abundant pollen grains were from *Prunus dulcis* and *Phacelia* spp., while managed *Nemophila* spp., wild grass species, and wild *Amsinckia* sp. comprised most of the pooled pollen in “other.”

*Osmia lignaria*‐foraging preferences for *Phacelia* spp. pollen changed significantly with both distance and time (Table 4). Pollen counts that favored almond pollen were concentrated in orchard interiors and during peak bloom. The inverse was observed for *Phacelia* spp. pollen grains in *O. lignaria* provisions, from which significantly higher detection of *Phacelia* spp. occurred closest to the wildflower planting and after peak almond bloom.

**Table 4:** ANOVA table for the proportional composition of pollen provisions sampled from completed *Osmia lignaria* nests

	Variable	*F*	*p*
Almond	Distance	5.78	.0045*
	Time	26.7	<.0001*
	Dist × time	2.27	.1094
Phacelia	Distance	4.15	.0191*
	Time	32.1	<.0001*
	Dist × time	0.56	.5754
Other	Distance	2.30	.1065
	Time	3.50	.0648
	Dist × time	1.72	.1881

Note

Pollen was collected during peak and postalmond bloom (time) at distances 85, 260, and 435 m from managed wildflower plantings. Data were analyzed separately for almond, *Phacelia*, and all “other” (pooled) pollen grains counted. For all analyses, *df* = (2, 84), (1, 82), and (2, 84) for distance, time, and dist × time, respectively.

* Significant at *p* ≤ .05

## 4 DISCUSSION

This study verified the critical role that access to alternative forage can have on managed *O. lignaria* nesting and reproductive success in commercial orchards. From the 2016 analysis of *O. lignaria* pollen provisions, we confirmed that nesting females collected pollen from the wildflower plantings from up to 800 m away. Most nest completion occurred at orchard edges (zones A1 and A2), and significantly, more cells were recovered from A1 over A2, which is likely a consequence of closer proximity to wildflower plantings. We conclude that planting and maintaining wildflowers is a promising strategy for integrating *O. lignaria* pollination into existing orchards, particularly in landscapes with limited alternative floral resources.

More progeny were recovered from nesting locations along orchard edges without adjacent forage plantings (zone A2; 410 m) than in the orchard interior, even when interior nests were proximally closer to the wildflower plantings (zones B1 and C1; 240 and 400 m, respectively). Even more remarkable was that no significant differences in nest completion over time were detected between A1 and A2 (both years), suggesting that, in this case, proximity to orchard edges is better predictors of managed *O. lignaria* nesting than relative proximity to alternative floral resources. The availability of open land with blooming wildflowers along orchard edges may have promoted bee nesting by mitigating some of consequences, for example, reduced floral diversity, of agricultural intensification in the area (Brodschneider & Carlsheim, 2010). Further, it is likely that almond blossoms and wildflowers along edges receive more sunlight and visibility, which could make them more attractive to foraging bees (Burgess, Kelly, Robertson, & Ladley, 2005). Additionally, it is well known that bees and other insects rely on visual landmarks for navigation across a given landscape (Collett & Collett, 2002). Possibly, abrupt orchard edges may have provided discrete visual cues for *O. lignaria* navigation, which promoted nesting near orchard edges. It is also critical to remember that although nesting over time did not vary between A1 and A2, significantly more viable cells were recovered from A1, which had the closest proximity to the plantings. This provides compelling evidence that the orchard edge combined with the short distance to the wildflower plantings synergized *O. lignaria* reproductive potential.

Nesting data collected over time demonstrate that although bloom ceased approximately 5 March in both years, nest completion continued at least 2 weeks beyond bloom, when only very limited floral resources (or none at all) were available outside of the wildflower plantings. At the same time, floral plot visitation data confirm that *O. lignaria* continued to access the wildflower plantings beyond almond bloom during both years. Polyfloral diets generally support bee performance better than a monofloral diet (Di Pasquale et al., 2013; Vaudo, Tooker, Grozinger, & Patch, 2015), and access to a variety of floral resources for foraging bees has been shown to support diverse and abundant bee communities across various agroecosystems. In this study, the importance of the wildflower plantings is highlighted by the exceptional increase in nesting over time observed at orchard edges and with proximity to the plantings. *Osmia lignaria* use of the plantings again is verified by the pollen composition of provision masses at peak almond bloom and after bloom. Overall, more *Phacelia* spp. pollen was found in sampled provisions closest to the wildflower plantings, and at all distances, *O. lignaria*‐foraging preferences shifted dramatically in favor of *Phacelia* spp. once almond blossoms became scarce. Importantly, we emphasize that orchard managers need not be concerned about reduced pollination efficiency to almond trees when *Phacelia* spp. is available. This is because of the regular and substantial incorporation of almond pollen into female *O. lignaria* provisions during bloom. In addition, while bees nesting closer to the floral enhancements incorporated relatively more *Phacelia* spp. into their provisions, it is also the case that those nearby zones also supported the highest rates of *O. lignaria* reproduction. Higher rates of female provisioning to *O. lignaria* progeny in nearby zones resulted in collectively greater pollen foraging than for females nesting farther away; this would compensate for any loss in pollination efficiency at the individual provision mass level.

It was not obvious whether the observed benefits of orchard edges are a consequence of bees released in orchard interiors migrating to nest boxes installed nearer the floral plantings, or because of increased individual fecundity of locally released females. Future work should explore whether migration or improved reproductive potential is underlying the pattern observed here.

Ongoing drought conditions may explain lower bee reproduction in 2015. It rained 1.8 mm during 2015 almond bloom and dry conditions continued in 2016, with only 12.4 mm rainfall at petal fall. Such dry weather likely restricted *O. lignaria* access to mud, which is required of females for nest construction and reproduction. The drought was further compounded by limited orchard irrigation during almond bloom. To curtail mud as a limiting resource, in 2016, water was distributed from tanks on operator‐owned trucks to orchard grounds where *O. lignaria* were nesting. In 2015, without mud artificially “made” for *O. lignaria* use during drought conditions, only 30% of the initial number of bees released into the orchards were recovered in the form of progeny to use for the following year. In 2016, 80% of released bees were recovered as progeny. More work is needed to identify ideal conditions and soil types for *O. lignaria* nest‐building, as artificial provisioning or augmentation of mud in orchards may facilitate successful nesting.

Many of the significant trends observed from progeny outcomes were not reproducible between years. For example, female‐to‐male ratios were significantly higher in zones further away from the wildflower plantings in 2016, while no such effect was observed in 2015. Female bees intrinsically have a higher pollination value, as they tend to live longer and need pollen and nectar resources for nest‐building, so understanding factors driving sex ratios in this system is important. Overall, sex ratios favored more females in 2015 versus 2016. Poorer overall nesting could bias sex ratios toward females, since female eggs are typically laid first in the nesting tunnel (Bosch & Kemp, 2001). This is the likely explanation for higher sex ratios in 2015, as fewer cells per tunnel were recorded in 2015 versus 2016. However, it does not explain why in 2016 sex ratios varied by zone, since cells per nesting tunnel were mostly consistent throughout all zones.

The low rates of predation and parasitism were fortunate. Because of managed incubation practices, *O. lignaria* are typically released into almond orchards prior to when wild populations of bees and their natural enemies are present. Thus, for growers wishing to employ *O. lignaria* in almonds, any treatment and processing of bees for parasitism and disease would require minimal consideration.

Unsurprisingly, *Phacelia* spp. was the dominant pollen type identified from completed *O. lignaria* provisions, since 96% of the wildflower plantings' floral area was provided by *Phacelia ciliata* (Lundin et al., 2017). While proportionally fewer *Nemophila* spp. flowers were available to foraging bees, it provided the only other managed pollen source that was discovered with regularity in pollen provisions. *Osmia lignaria* reproduction in 2016 far exceeded what has been achieved by researchers in previous years (typical 30%–40% female bee return; Artz, Allan, Wardell, & Pitts‐Singer, 2013, 2014, vs. 80% return in 2016). This makes us question the value other managed wildflower species had in supporting *O. lignaria* populations. Perhaps future seed mixes customized to support *O. lignaria* in orchards should concentrate solely on planting *P. ciliata* as alternative forage, rather than looking to more diverse and potentially more costly seed mixes. However, any potential benefit of other wildflower species cannot be completely ruled out, considering *O. lignaria* may rely on other wildflower species for nectar, which comprises a substantial proportion by weight of a typical *O. lignaria* provision.

Materials presented in Appendix S2 and Table S6 demonstrate that in extreme cases of resource limitation, *O. lignaria* females will fly at least 1,600 m round‐trip to build pollen provisions. It is an astonishing distance for *O. lignaria* to travel, considering the amount of time and energy required to access alternative pollen sources, and the availability of vacant nesting sites in zones that are closer in proximity to managed wildflower plots. Rust (1990) determined that *O. lignaria*‐foraging distances generally fall within 600 m of their nest site, and we demonstrate here that their foraging range will exceed that distance if necessary.

We propose that orchardists wishing to employ *O. lignaria* pollination in their own production systems will maximize their success with the installation of nearby, coblooming wildflower plantings. Currently, the largest expense associated with *O. lignaria* management is costs associated with bee acquisition—retail costs of *O. lignaria* are typically upwards of $1.50 (USD) per individual female (J. Watts, personal communication). Comparatively, honeybee hive rentals typically cost ca. $190 (USD) per colony for California almond growers to rent during bloom (Goodrich, 2018). With a stocking rate of 750 *O. lignaria* females per hectare required for almond pollination, we must identify management practices that favor the highest possible retention of nesting females and rates of in‐orchard bee propagation to reduce annual pollination costs. This would also confer the greatest benefit to wild ecosystems, as it would decrease the industry's reliance on harvesting *O. lignaria* cocoons from native wildlands (Tepedino & Nielson, 2017), where consequential ecological impacts are largely unknown.

Further, wildflower plots do not need to be large to benefit nesting *O. lignaria* and other bees. Occupying just 0.48 ha each, our plots required a relatively small commitment of land, labor, and resources compared to the vast acreage of almonds grown in the vicinity. Most postalmond bloom nesting occurred within zones along orchard edges. The beneficial effects of the wildflower plantings in aiding *O. lignaria* reproduction were less obvious in orchard interiors. Future studies to investigate the impacts of alternative forage on *O. lignaria* propagation should consider interspersing small wildflower plots regularly throughout managed orchards, as the benefit to nesting populations diminished toward the orchard interior. This may provide more accessible alternative forage to bees nesting deep within larger orchard blocks. Regardless, it is apparent that the availability of any coblooming alternative floral resources can facilitate *O. lignaria* success in commercial almond orchards, especially when adequate mud is available for female nest construction.

## CONFLICT OF INTEREST

None declared.

## AUTHOR CONTRIBUTIONS

All authors contributed to conception, ideas, and designed methodology; NKB, DRA, OL, KW, DP, and TLP‐S collected the data; NKB and OL analyzed the data; NKB led the writing of the manuscript. All authors contributed critically to the drafts and gave final approval for publication.

## Data Availability

All raw data analyzed and presented in this manuscript, along with photographs of all pollen identification “type” slides, have been uploaded and made freely available on the Pennsylvania State University Libraries archiving website, ScholarSphere (https://doi.org/10.26207/zmrd-hr29).
