# Supplementary material for: Wildflower plantings promote blue orchard bee, Osmia lignaria (Hymenoptera: Megachilidae), reproduction in California almond orchards
Source: Ecol Evol. 2020 Feb 25;10(7):3189–99. doi: 10.1002/ece3.5952 (PMC7141036; doi:10.1002/ece3.5952)
Supplement: Supplementary file 1 [file ECE3-10-3189-s001.docx]

**Appendix S1**

**2015 & 2016 BLUE ORCHARD BEE RELEASE PRACTICES**

Approximately five days prior to bloom, bees were incubated at 28° C to facilitate timely adult emergence and released at 15% almond bloom. Almond bloom started on 12 February 2015 and 13 February 2016, respectively. In 2015, emerging *O. lignaria* were released in enclosed wooden stands measuring 46 x 40.5 x 69 cm (Fig S1a). In 2016, *O. lignaria* were instead released in Styrofoam containers suspended from almond branches in the orchards (25.5 x 40 x 30.5 cm; Fig S1b). At the time of their release, approximately 20% of adult *O. lignaria* had already emerged from their cocoons. To ensure effective delivery of the remaining unemerged adults, all emergence boxes were removed nightly from their representative zone and stored in a propane-powered mobile incubator (Fig S1c; temperature held at a near-constant 28° C) (2015) or in a temperature-controlled incubator off-site (at 29° C; 2016) for the first three nights following their release and returned to their respective release sites early the following morning.

**Appendix S2**

BLUE ORCHARD BEE VISITATION PROTOCOL (adapted from Lundin et al., 2017)

The observer slowly walked along each transect for 10 minutes of active observation twice during each sampling timepoint. During this time, the observer counted *O. lignaria* landing on reproductive structures of flowers that fell within 1m of one side of each transect (side of transect was selected at random). The alternate side of the transect was sampled during the second walk/observation period. *O. lignaria* observations were summed across the five sampling time points in wildflower and fallow plots by year prior to statistical analyses.

**References**

Lundin, O., Ward, K. L., Artz, D. R., Boyle, N. K., Pitts-Singer, T. L., & Williams, N. M. (2017) Wildflower plantings do not compete with neighboring almond orchards for pollinator visits. Environmental Entomology, 46, 559 – 564. doi: 10.1093/ee/nvx052.

**Appendix S3**

LONG DISTANCE SITE: NESTING OVER TIME AND POLLEN PROVISION COMPOSITION

In 2016 only, three replicated sets of ten uniformly-spaced nest boxes each were installed at the far end of orchard blocks supporting zones A1 – C1. These nest boxes were located approximately 800 m away from the wildflower planting and received a limited release of approximately 475 female and 800 male *O. lignaria* cocoons in each of the three sections (each 0.86 ha). The purpose of these ‘long distance’ (‘LD’) nest sites were to evaluate whether *O. lignaria* would continue to benefit from the floral plantings at extreme distances that move well beyond their reported foraging range of 600 m from the nest site (Rust, 1974).

Counts of plugged tunnels were also made for the LD nest boxes (methods are detailed in the main body of the manuscript), although due to differences in nest box placement and the numbers of bees released, this dataset was interpreted and analyzed separately from other nesting data.

Pollen provision composition of completed 2016 LD nests were determined in completed with the methods described in the main body of the manuscript. We sampled every plugged nest at the end of bloom to assess pollen provision composition. Long distance pollen sampling occurred after *O. lignaria* nesting ceased, and rows with completed nests were not painted as described earlier for nests collected from zones A-C. Therefore, no temporal component was incorporated into LD nest site analysis (*i.e.*, peak versus post-bloom).

At the 2016 LD sites, much less nesting was observed compared to closer orchard edges and wildflower plantings, and nesting stalled almost completely after bloom (Fig S2). In total, 119 completed nests were removed from the LD boxes and examined for pollen provision composition. 49.6% of the LD provisions included *Phacelia* *spp.* pollen grains. Overall, *Phacelia spp.* pollen occupied 20.2% of the total volume of pollen evaluated at long-distance sites. A detailed breakdown of the most common pollen grains detected in LD nests as are presented in Table S6.

**Appendix S**4

***Within-zone pollen provision composition: volume-adjusted data***

Since almond pollen grains are much larger in size that any other pollen grains encountered in the provision, percent by volume was determined for each provision by calculating the average dimensions of the dominant pollen species (10 grains each of *P. dulcis* and *Phacelia* spp.) and extrapolating average pollen grain volume from those metrics (Buchmann and O’Rourke 1991, da Silveira 1991). Due to the limited abundance and variability in size of alternative pollen types detected, only counts from *P. dulcis* and *Phacelia* spp. almond pollen were volume-adjusted and subsequently considered for statistical analysis. Data was analyzed via a general linear model with season (peak versus post bloom) and distance as fixed effects in SAS.

After correcting for variability in pollen grain size (and eliminating ‘other’ pollen grains from the counts), we continued to see a significantly lower volume of *Phacelia* spp. pollen in provision masses completed at peak versus late almond bloom (F(1,1) = 51.07, *P* < 0.0001). Additionally, the volume of *Phacelia* spp. pollen in *O. lignaria* provisions decreased with distance from the wildflower plantings (F(2,2) = 5.56, *P* = 0.0054). Overall, on a per-nest basis, the categorically dominant pollen type per provision sampled changed from 68% almond and 32% *Phacelia spp.* at peak bloom to 14% almond and 86% *Phacelia spp.* post-bloom using volume-adjusted data.

**References:**

**da Silveira, F. (1991)** Influence of pollen grain volume on the estimations of the relative importance of its source to bees. Apidologie, 22, 495 – 502. doi: 10.1051/apido:19910502

**Buchmann, S. L., & O’Rourke, M. K. (1991)** Importance of pollen grain volumes for calculating bee diets. Grana, 30, 591 – 595. doi: 10.1080/00173139109427817


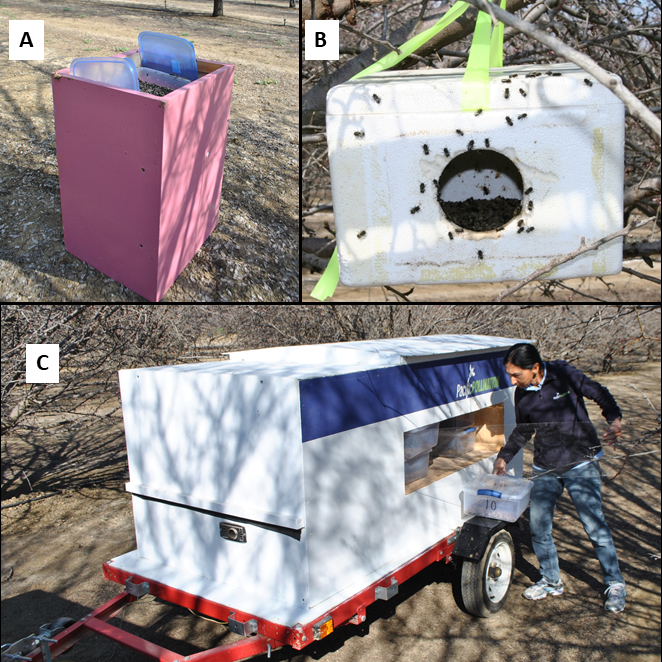


Figure S1. Materials for managing large populations of *O. lignaria* for commercial pollination include (a) 2015 *O. lignaria* release boxes, (b) suspended Styrofoam release boxes used in 2016, (c) a propane-powered, temperature-controlled holding bay to accelerate overnight *O. lignaria* emergence


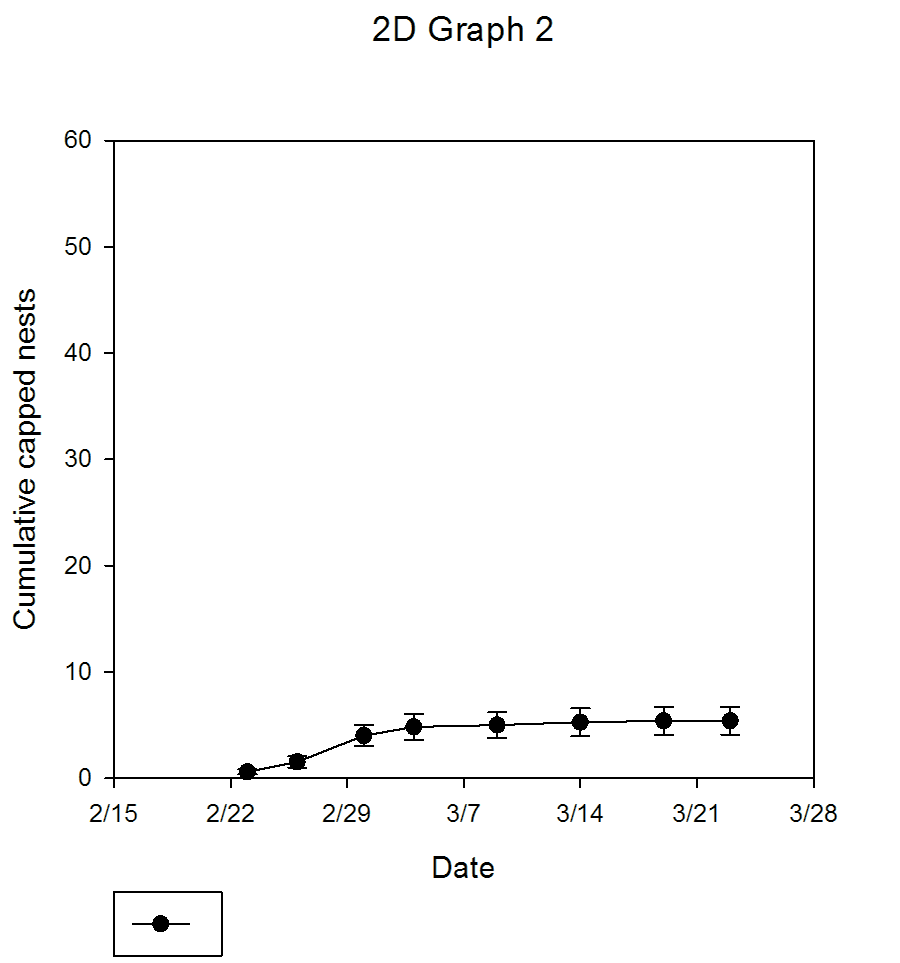


Figure S2. Mean *O. lignaria* nest completion over time, ± SEM, in 2016 for n = 30 total nest boxes (10 per replicate) located 800m away from wildflower plantings.

Table S1. ANOVA table for *Osmia lignaria* nesting over time in a California almond orchard, 2015 and 2016. Model was constructed using a normal distribution with an identity link function for additive completed nests over time with replicate as a random effect.

|  | **2015** | | | **2016** | | |
| --- | --- | --- | --- | --- | --- | --- |
| **Effect** | **DF** | **F** | **P** | **DF** | **F** | **P** |
| Zone | 5,12 | 4.73 | 0.0129* | 5, 10 | 18.47 | <0.0001* |
| Date | 5, 60 | 130.31 | <0.0001* | 7, 84 | 96.54 | <0.0001* |

Table S2. Tukey comparisons for nesting over time, by zone. For all comparisons, 2015 and 2016 df = 10 and 12, respectively

|  |  | 2015 | | 2016 | |
| --- | --- | --- | --- | --- | --- |
| Zone 1 | Zone 2 | t | Adj *P* | t | Adj *P* |
| A1 | B1 | 2.94 | 0.1 | 4.84 | **0.0066*** |
| A1 | C1 | 3.34 | 0.052 | 6.61 | **0.0006*** |
| A1 | A2 | 1.32 | 0.771 | 2.07 | 0.3703 |
| A1 | B2 | 3.85 | **0.022*** | 7.50 | **0.0002*** |
| A1 | C2 | 3.71 | **0.0278*** | 7.09 | **0.0003*** |
| B1 | C1 | 0.40 | 0.998 | 1.77 | 0.1064 |
| B1 | A2 | -1.62 | 0.602 | -2.76 | 0.1449 |
| B1 | B2 | 0.91 | 0.937 | 2.66 | 0.1683 |
| B1 | C2 | 0.77 | 0.968 | 2.25 | 0.2956 |
| C1 | A2 | -2.02 | 0.384 | -4.54 | **0.0101*** |
| C1 | B2 | 0.50 | 0.995 | 0.89 | 0.9415 |
| C1 | C2 | 0.36 | 0.999 | 0.48 | 0.996 |
| A2 | B2 | 2.53 | 0.19 | 5.42 | **0.0003*** |
| A2 | C2 | 2.39 | 0.234 | 5.01 | **0.0051*** |
| B2 | C2 | -0.14 | 1 | -0.41 | 0.998 |

Table S3. ANOVA table for progeny outcomes of all *O. lignaria* cells recovered from commercial almond orchards, 2015 and 2016. One-way ANOVAs with replicate as a random factor evaluated differences in *O lignaria* reproduction by zone in SAS PROC ANOVA. Tukey’s multiple comparisons were conducted to distinguish differences between zones (pictured in Fig. 5).

|  | **2015** | | | **2016** | | |
| --- | --- | --- | --- | --- | --- | --- |
| **Variable** | **DF** | **F** | **P** | **DF** | **F** | **P** |
| Live cells per nest box | 5 | 3.324 | 0.0056* | 5 | 64.41 | <0.0001* |
| Proportion cell mortality | 5 | 33.475 | <0.0001* | 5 | 4.219 | 0.0009* |
| Sex ratio (F:M) | 5 | 1.305 | 0.2596 | 5 | 11.05 | <0.0001* |
| No. cells/tunnel | 5 | 2.3911 | 0.0365* | 5 | 4.138 | 0.001* |
| Proportion died in development | 5 | 3.347 | 0.0054* | 5 | 9.171 | <0.0001* |
| Proportion pollen ball | 5 | 1.231 | 0.2926 | 5 | 4.352 | 0.0007* |

Table S4. 2015 summary of average cell outcome per nestbox (100 nesting tunnels per nestbox; 40 nest boxes per zone), by zone (± SEM). Presented with zone is the average relative distance to maintained wildflower plantings (m). Each mean value is followed by the relative percentage of cells recovered at that stage for each respective zone.

| Zone | X̄ cells/box | Live ♀ | Live ♂ | Pollen ball^a^ | Scav/Para^b^ | Died Dev^c^ |
| --- | --- | --- | --- | --- | --- | --- |
| A1 (80 m) | 72.4 ± 5.3 | 25.6 ± 1.9 (35%) | 33.1 ± 2.9 (46%) | 9.1 ± 0.9 (13%) | 1.7 ± 0.3 (2%) | 4.3 ± 0.5 (5%) |
| B1 (240 m) | 32.0 ± 3.9 | 11.8 ± 1.4 (37%) | 14.5 ± 2.0 (45%) | 3.6 ± 0.6 (11%) | 1.0 ± 0.2 (3%) | 2.0 ± 0.2 (6%) |
| C1 (400 m) | 32.2 ± 3.9 | 12.2 ± 1.4 (37%) | 13.9 ± 1.9 (42%) | 3.7 ± 0.6 (10%) | 1.2 ± 0.2 (3%) | 2.2 ± 0.1 (7%) |
| A2 (410 m) | 51.4 ± 5.2 | 16.1 ± 1.4 (30%) | 25.0 ± 2.9 (47%) | 6.4 ± 1.0 (12%) | 1.9 ± 0.3 (3%) | 3.8 ± 0.7 (7%) |
| B2 (475 m) | 31.3 ± 4.4 | 10.0 ± 1.3 (30%) | 15.0 ± 2.2 (46%) | 4.0 ± 0.8 (13%) | 1.3 ± 0.2 (3%) | 2.3 ± 0.2 (7%) |
| C2 (580 m) | 35.7 ± 4.0 | 12.4 ± 1.5 (34%) | 16.3 ± 2.0 (44%) | 4.3 ± 0.6 (11%) | 1.2 ± 0.2 (3%) | 2.5 ± 0.2 (7%) |

^a^Pollen ball occurs when the provision remains uneaten in the cell, likely due to egg failure.

^b^Scav/Para refers to cells that failed to develop due to scavenger and parasite activity (confirmed by X-radiography).

^c^Died dev includes all cells that failed to develop to become viable adults for unknown reasons. This column pools larval and pupal mortality, as confirmed by X-radiography.

Table S5. 2016 summary of average cell outcome per nestbox (100 nesting tunnels per nestbox; 40 nest boxes per zone), by zone (± SEM). Presented with zone is the average relative distance to maintained wildflower plantings (m). Each mean value is followed by the relative percentage of cells recovered at that stage for each respective zone.

| Zone | X̄ cells/box | Live ♀ | Live ♂ | Pollen ball^a^ | Scav/Para^b^ | Died Dev^c^ |
| --- | --- | --- | --- | --- | --- | --- |
| A1 (80 m) | 324.0 ± 24.4 | 64.9 ± 5.3 (20%) | 167.8 ± 12.7 (52%) | 58.7 ± 5.7 (18%) | 5.0 ± 0.8 (2%) | 27.6 ± 3.6 (9%) |
| B1 (240 m) | 169.1 ± 16.1 | 36.7 ± 3.5 (22%) | 87.2 ± 8.6 (52%) | 26.2 ± 3.0 (16%) | 2.8 ± 0.4 (2%) | 16.0 ± 3.6 (9%) |
| C1 (400 m) | 136.5 ± 17.6 | 28.2 ± 3.7 (21%) | 66.1 ± 8.8 (48%) | 23.3 ± 3.4 (17%) | 3.2 ± 0.6 (2%) | 15.7 ± 3.8 (12%) |
| A2 (410 m) | 238.8 ± 20.6 | 42.7 ± 3.5 (18%) | 101.4 ± 8.9 (42%) | 48.7 ± 5.4 (20%) | 3.4 ± 0.5 (1%) | 42.5 ± 5.9 (18%) |
| B2 (475 m) | 97.5 ± 10.5 | 23.6 ± 2.8 (24%) | 40.4 ± 4.8 (41%) | 14.9 ± 1.8 (15%) | 1.7 ± 0.2 (2%) | 17.1 ± 3.7 (18%) |
| C2 (580 m) | 120.6 ± 11.5 | 28.1 ± 2.9 (23%) | 49.6 ± 5.2 (41%) | 20.4 ± 2.2 (17%) | 2.0 ± 0.3 (2%) | 20.5 ± 2.9 (17%) |

^a^Pollen ball occurs when the provision remains uneaten in the cell, likely due to egg failure.

^b^Scav/Para refers to cells that failed to develop due to scavenger and parasite activity (confirmed by X-radiography).

^c^Died dev includes all cells that failed to develop to become viable adults the next year, for unknown reasons. This column pools larval and pupal mortality, confirmed by X-radiography).

Table S6. Mean pollen provision composition (% ± SEM) of all completed nests at long distance sites. Only plant species comprising at least 1% of the average provision are listed here.

| Species | Mean % pollen, ± SEM |
| --- | --- |
| *Prunus* | 74.2 ± 2.9 |
| *Phacelia* | 16.8 ± 2.3 |
| *Nemophila* | 2.2 ± 0.7 |
| *Amsinckia* | 1.1 ± 0.2 |
| Grass | 1.9 ± 0.2 |
